# Short Report: Comparison of three methods for identifying implementation determinants to measurement-based care

**DOI:** 10.21203/rs.3.rs-7973723/v1

**Published:** 2025-11-19

**Authors:** Ruben G. Martinez, E. Ruby Cramer, Lesley A. Norris, Abigail Matson, Christian Lang, Nora B. Henrikson, Paula R. Blasi, Lorella G. Palazzo, Andrea J. Hoopes, Shannon Dorsey, Bryan J. Weiner

**Affiliations:** Warren Alpert Medical School of Brown University: Brown University Warren Alpert Medical School; Brown University Warren Alpert Medical School; Brown University Warren Alpert Medical School; Kaiser Permanente Washington Health Research Institute; Brown University Warren Alpert Medical School; Kaiser Permanente Washington Health Research Institute; Kaiser Permanente Washington Health Research Institute; Kaiser Permanente Washington Health Research Institute; Kaiser Permanente Washington Health Research Institute; University of Washington; University of Washington

**Keywords:** implementation determinants, rapid ethnography, rapid evidence synthesis, measurement-based care, design probes

## Abstract

**Background:**

Identifying implementation determinants, also referred to as barriers and facilitators, is considered a critical component of implementation science. There are many emerging methods for identifying implementation determinants, yet very few evaluations of how these methods complement or diverge from one another. The objective of this report is to compare three methods for identifying determinants in the context of the Novel Methods for Implementing Measurement-Based Care with Youth in Low-Resource Environments study.

**Methods:**

Rapid evidence synthesis involves a targeted review of empirical literature. Rapid ethnographic assessment uses site visits, interviews, and observations to develop an insider’s perspective. Design probes engage participants in prompted activities (e.g., journaling, taking photos) to surface insights from their lived experience. We compared convergence of determinants identified by each method using a Jaccard plot and pairwise Jaccard indices.

**Results:**

All three methods combined produced a list of 42 determinants. Rapid evidence synthesis surfaced 29 (69%) determinants, including 8 solely identified by this method. Rapid ethnographic assessment surfaced 35 (83%) determinants, with 4 solely identified by this method. Design probes surfaced 23 (66%) determinants and did not surface any unique determinants. A total of 14 (33%) determinants were identified by all methods. Pairwise Jaccard indices indicated the strongest convergence between rapid ethnographic assessment and design probes (*J* = .66) and rapid evidence synthesis and rapid ethnographic assessment (*J* = .52). Convergence between rapid evidence synthesis and design probes (*J* = .37) was more modest.

**Discussion:**

This study describes the convergence of implementation determinants surfaced using three methods. We found substantial overlap between methods, with one third of determinants surfaced by all three methods. Despite this overlap, each method added unique insights. Rapid evidence synthesis surfaced determinants from the literature and theory that were less likely to be identified by rapid ethnographic assessment or design probes. Rapid ethnographic assessment had the highest yield of determinants. Design probes highlighted participant-driven perspectives that overlapped substantially with rapid ethnographic assessment; notably, design probes did not surface any new determinants. These results reinforce the complementary nature of multi-method determinant assessment while highlighting tradeoffs researchers must weigh when selecting determinant identification methods.

**Trial registration:**

Clinicaltrials.gov. NCT05644756. Registered 11/18/2022. This trial was retrospectively registered, https://classic.clinicaltrials.gov/ct2/show/NCT05644756

## Background

Identifying implementation determinants is an important step in designing contextually-relevant, targeted implementation strategies.^1^ However, common approaches to assessing determinants often lack the nuance necessary to capture the complexity of how determinants are functioning in the context of a clinic. Reviews suggest that the most common approach to determinant assessment involves key informant interviews and/or focus groups conducted with a limited subset of constituents (e.g., administrators).^2^ This emphasis on feasibility can overlook key determinants that are most strongly linked to implementation success.^3^ These approaches also rarely integrate determinants from the literature base, and are subject to limitations of constituent recall, bias, and issues of social desirability.^4^

In response to these challenges, a range of innovative methods to rapidly and comprehensively identify determinants have emerged. Rapid evidence synthesis (RES) allows for the integration of theory-based determinants by systematically identifying and synthesizing relevant peer-reviewed literature on a relatively rapid timeline.^5–7^ Second, rapid ethnographic assessment (REA) is an intensive ethnographic approach that leverages observations and interviews with a range of informants to develop an “insider’s perspective” of the implementation context.^8^ Third, design probes (or probes) center participants’ lived experience by collecting real-time data via journals, disposable cameras, and other prompted activities to surface participant-driven insights on implementation challenges that may not emerge in individual interviews.^9, 10^ Each method offers unique advantages: RES has the potential to harness and allow for comparisons to the broader literature base, REA is a powerful tool for understanding behaviors and determinants in context, and probes ground in constituent perspectives and experiences. Yet, little is known about the extent to which determinants identified using these methods converge or diverge, leaving researchers without guidance on which method to select. This limits the translation of such methods from research to practice, where the benefit of nuance that comes with multi-method assessment approaches must be balanced with resource availability, particularly in under-resourced settings.

This short report evaluates the convergence of determinants identified through RES, REA, and probes in the context of the NIMH-funded IMPACT Center’s Novel Methods for Implementing Measurement-Based Care with Youth in Low-Resource Environments (NIMBLE) study.^11^ By evaluating the convergence of determinants identified by each method descriptively and quantitatively, we aim to provide practical guidance on how each method contributes to determinant identification.

## Methods

### NIMBLE Overview

The NIMBLE study sought to optimize clinician’s use of measurement-based care in 4 community mental health centers (CMHCs) in Washington state. Measurement-based care (MBC) is an evidence-based practice for improving engagement and shared decision-making in youth mental healthcare by conducting regular assessments of clinical progress and sharing that information with clients.^12^ Clinics, clustered into two cohorts (study protocol),^11^ were each involved with NIMBLE for 18 months between 2021–2023.

## Study Design

### Rapid Evidence Synthesis (RES)

RES are tightly scoped, systematic reviews of literature. RES has gained some traction in recent years because it enables researchers to synthesize evidence on a timeline that is aligned with the needs of fast-paced implementation research.^7, 13^ The RES, guided by the IMPACT Center toolkit, sought to describe the known determinants to implementing MBC in under-resourced youth behavioral or mental health care settings. This search began April 1, 2022. Synthesis was completed on May 31st, 2022.

#### Search Strategy.

**Figure 1:**
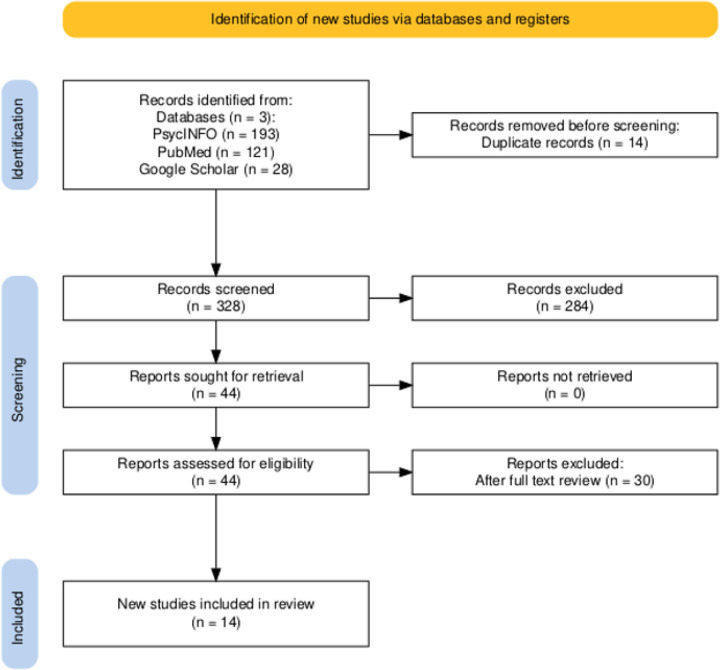
PRISMA flowchart for the rapid evidence synthesis

RES included a search of PubMed and PsycINFO as well as a rolling review of Google Scholar. The lead author screened titles and abstracts. Two team members trained in systematic review methods independently completed full-text review. Discrepancies were resolved by consensus. Eligible articles were English-language, quantitative, qualitative, or mixed-methods studies reporting on barriers/facilitators to the use of MBC with youth in community mental/behavioral health settings between 2018–2022. In total, 342 articles were identified by the search. The final synthesis included 14 articles (see Fig. 1 for the PRISMA diagram). Additional File 1 includes search strings, eligibility criteria, and included studies.

#### Data Abstraction and Synthesis.

Determinants and other study data were abstracted by the first author. To synthesize determinants, the first author extracted direct quotes from each paper into a list of “determinant statements.” The research team iteratively synthesized these determinant statements into themes. Initial themes were derived from a past narrative review on MBC determinants.^14^

### Rapid Ethnographic Assessment (REA)

REA explores the “insider’s perspective” of an implementation context.^8, 15^ Our REA was guided by the IMPACT Center toolkit, and its purpose was to understand each clinic’s context: to understand how determinants were operating within the context and to surface additional determinants to MBC.

#### Participants.

**Figure 2:**
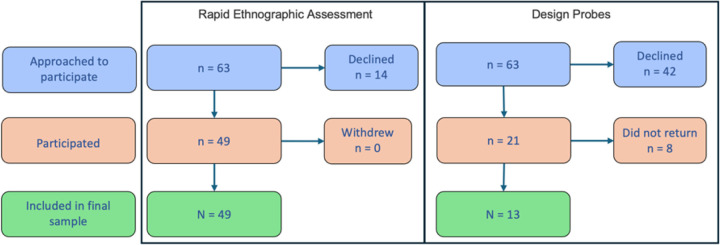
Participant Flow Diagram for Rapid Ethnographic Assessment and Design Probes in NIMBLE *Note*. NIMBLE = Novel Methods for Implementing Measurement-Based Care in Low-Resource Environments.

REA was conducted with *N* = 49 participants. A total of *n* = 30 identified as clinicians or trainees, *n* = 9 were supervisors or clinical leaders, *n* = 2 were information management/quality improvement specialists, and *n* = 8 were administrative staff (e.g., records and registration). See Fig. 2 for a participant flow diagram.

#### Site Visits.

Ethnographic visits lasted between 1.5–2 days at each site, including: (1) 15–30 minute interviews, and (2) walkthroughs or observations of clinic spaces and processes. The first author developed a dossier of each clinic and structured positionality exercises, which the research team engaged in before, during, and after each ethnographic visit. Interviews were audio-recorded, as were debriefing sessions with the research team. The interview guide was informed by the RES findings and developed in collaboration with the IMPACT methods core, which included two clinical experts in MBC. The interview guide included questions about: (1) the individual’s knowledge of MBC as a practice, (2) determinants to using MBC at their clinic, (3) their current use and anticipated modifications needed to MBC to be used in their clinic, (4) current or needed organizational supports for using MBC, (5) perception of a “shared vision” for MBC in their organization. We determined that saturation had occurred when debrief discussions confirmed that multiple team members’ perspectives were aligned. All three members of the research team took detailed field notes. All audio recordings were transcribed, de-identified, and cleaned. Team debriefs and written notes were turned into a single text corpus, organized by time.

#### Distillation and Synthesis.

We used a framework matrix approach for rapid qualitative analysis.^16^ Two coders independently read each episode (interview, meeting, or field note), copying exact quotes into a spreadsheet, where each column corresponded with an interview question. The team met again to resolve discrepancies in each episode to ensure completeness and consistency of extracted information. Following this, each coder independently summarized each determinant statement into a shorter statement. The team met following this step to synthesize these shorter statements into determinants using the themes identified in RES, iteratively separating or combining themes as new evidence emerged. REA analysis was completed within 3 months of site visits.

### Design Probes

Design probes (probes) are a novel application of a participatory research method wherein participants engage in prompted activities (e.g., journaling, taking photos) to surface insights from their lived experience.^10^ Probes were used in NIMBLE to identify determinants to the implementation of MBC.

#### Participants.

Probes were completed by *N* = 13 participants (Fig. 2). A total of *n* = 12 were clinicians and *n* = 1 was a supervisor/clinical leader.

#### Probe Structure.

Probes included a journal, pen, 7 activity prompts that asked about different aspects of MBC (e.g., physical space), a stress ball, and a snack (2 pieces of chocolate). All 7 design activity prompts can be viewed in Additional File 2.

#### Synthesis.

One research team member transcribed written text from journals into Microsoft Word. Two coders independently developed summary memos that outlined key points verbatim. Following the summary, the team met to synthesize these statements into determinants using the themes identified in RES and REA, iteratively separating or combining themes as new evidence emerged. Probe analysis was completed within 3 months of site visits.

### Analytic Plan: Evaluating Method Convergence

Determinants were categorized by the Consolidated Framework for Implementation Research (CFIR) domains through consensus discussions.^17^ We compared overlap in determinants identified by each method in each CFIR domain using a Jaccard index.^18^ To determine similarity of identified determinants between methods, we calculated pairwise Jaccard indices, using the equation: $J=\frac{C}{N'}$, where *C* is the number of determinants identified by both methods and *N’* is the total number of determinants.^18^ We felt that this was the most appropriate way to compare methods, as each method had a different number of inputs (people vs. articles). To do this, we generated a spreadsheet with a binary indicator (1/0) of whether the determinant was surfaced by the method.

## Results

The synthesis of all three methods resulted in 42 total determinants. Determinants were identified at the levels of individual patient/client (15; 36%), inner setting (14; 33%), implementation process (5; 12%), innovation (7; 17%), and outer setting (1; 2%). Fourteen determinants (33%) were identified by all methods. Table 1 displays all determinants cross-referenced with each method that surfaced the determinant. Of the 14 determinants identified by all methods, 4 at the levels of the individual patient or clinician, 4 at the inner setting, 2 at the implementation process, and 4 at the innovation. These determinants, as organized by ecological level, were:

- Individual (patient/clinician)
  - Patient/client resistance to or concerns with completing measures
  - Clinician/staff attitudes toward measures, MBC
  - Clinician knowledge, self-efficacy, and skill
  - Competing demands/lack of time in session
- Inner setting
  - Organizational norms, culture, and climate
  - General administrative burden (competing demands outside of session)
  - Lack of built-in time to do administrative MBC (e.g., review scores)
  - Lack of reminders
- Implementation Process
  - Lack of centralized policies, procedures, and communications related to MBC
  - No initial or continued training on measures, the MBC process, and MBC system
- Innovation
  - MBC system or workflow is cumbersome to patients and/or clinicians
  - Cultural and linguistic relevance of measures
  - Clinicians and/or patients cannot easily access measure scores/item scores/clinical cutoffs
  - Complexity of MBC as a clinical practice

### Rapid Evidence Synthesis

RES alone surfaced 29 determinants (69%). Determinants were surfaced at the level of the individual patient/clinician (11), inner setting (10), implementation process (3), 4 innovation (4), and outer setting (1). Eight determinants were identified solely by RES:

- Individual (patient/clinician)
  - Insurance status
  - Attendance
  - Turnover
  - Clinicians decide who does/does not get MBC
  - Clinical presentation
- Inner setting
  - Clinics lack technological infrastructure to support MBC or MBC program
  - Low baseline MBC use
- Outer setting
  - Organizations do not have guidance on selecting standardized self-report measures

### Rapid Ethnographic Assessment

REA alone surfaced 35 (83%) determinants. Eleven determinants were surfaced at the levels of the individual clinician/patient, the inner setting (12), implementation process (5), and the innovation (7). Four determinants were solely identified by REA:

- Individual patient/clinician
  - Youth patients do not want to involve parents in treatment
- Inner Setting
  - No discussion within or between teams about MBC process
- Implementation Process
  - Therapists do not receive feedback on MBC (supervision or administrative)
- Innovation
  - Frequent changes to MBC process

### Design Probes

Probes alone surfaced 23 (66%) determinants. Seven were at the level of the individual patient/clinician, 7 at the inner setting, 3 at the implementation process, and 6 at the innovation. Probes did not surface any unique determinants.

### Similarity of Determinants Across Methods

**Figure 3:**
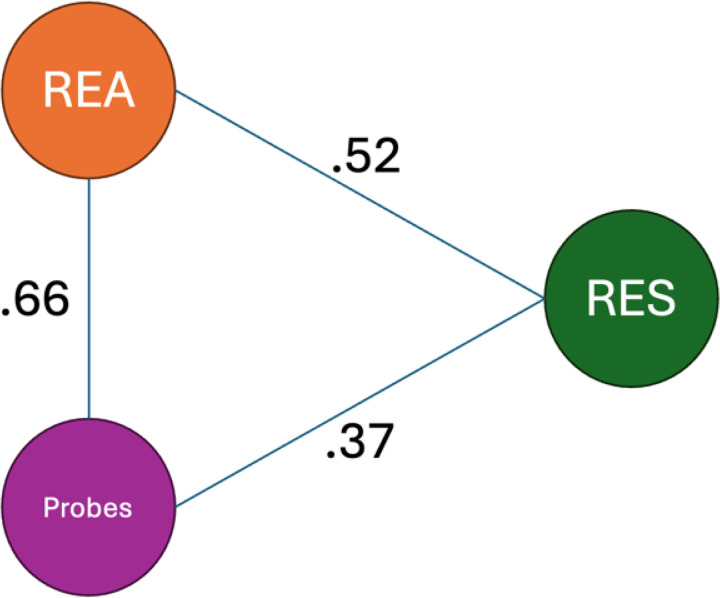
Jaccard Network Diagram Representing the Convergence of Barrier Identification Methods in NIMBLE *Note*. NIMBLE = Novel Methods for Implementing Measurement-Based Care in Low-Resource Environments; REA = Rapid Ethnographic Assessment; RES = Rapid Evidence Synthesis; DP = Design probes.

**Figure 4:**
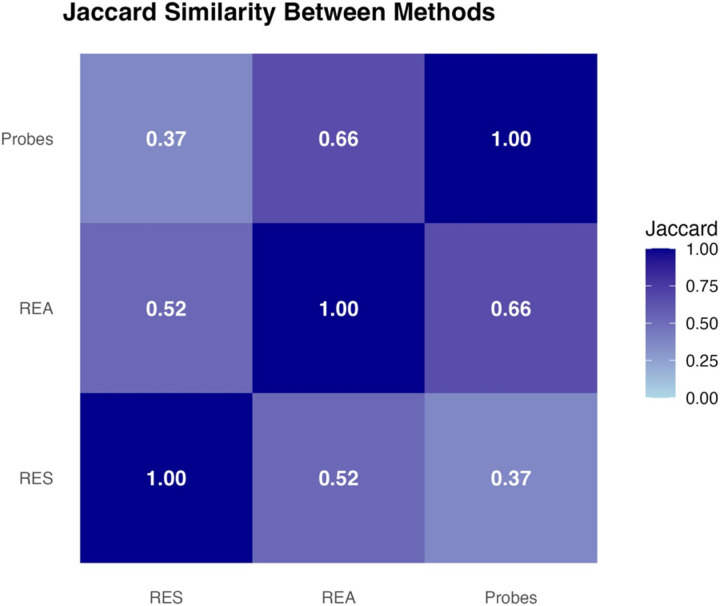
Heatmap Representing the Convergence of Barrier Identification Methods in NIMBLE *Note*. NIMBLE = Novel Methods for Implementing Measurement-Based Care in Low-Resource Environments; REA = Rapid Ethnographic Assessment; RES = Rapid Evidence Synthesis; DP = Design probes.

Pairwise Jaccard indices indicated the strongest overlap between REA and probes (*J* = .66), suggesting that the two methods that drew from lived experience and observation captured many of the same determinants. The second strongest overlap was between REA and RES (*J* = .52), indicating that about half of the determinants identified by each method were not identified by the other. Overlap between RES and probes (*J* = .37) was more modest, reflecting that RES and probes surfaced a somewhat unique set of determinants. See Figure 3 for the Jaccard Plot and Figure 4 for a heatmap representing the overlap in determinants across methods.

## Discussion

This study describes the convergence of implementation determinants surfaced using three innovative methods: Rapid ethnography, rapid evidence synthesis, and design probes. We found substantial overlap between methods, with one third of determinants surfaced by all three methods. Despite this overlap, we also found that each method added unique insights. RES surfaced determinants from the literature and theory that were less likely to be identified by REA or probes. REA had the highest yield of determinants, identifying the majority (83%) of determinants. Probes highlighted participant-driven perspectives that overlapped substantially with REA; notably, probes did not surface any new determinants but appear to be a viable substitute for REA when in-person observations are not possible. Pairwise Jaccard analysis highlighted these patterns, with the strongest similarity between REA and probes, and more modest overlap between RES and the other two methods.

### Implications for Implementation Research

Selecting which determinant identification methods to use has important implications for resources (e.g., staffing, budget, time). These findings suggest that relying on one method may miss key determinants, and that incorporating more than one method results in a richer yield of results. REA and probes, which center on understanding lived experience through observation and interactive exercises, yield deep contextual insights. On the other hand, RES appears valuable for surfacing determinants that are not surfaced in the field. These results reinforce the complementary nature of multi-method assessment while highlighting tradeoffs researchers must weigh when selecting determinant identification methods.

### Recommendations for Method Selection

All three methods were feasible to complete over a time frame of 3 months. When resources are limited, REA appears to provide the greatest “bang for the buck,” surfacing the largest number of determinants and yielding deep contextual insights. Probes may serve as a suitable alternative to REA in contexts or with populations where it is not possible to do an in-person or virtual visit. RES appears to surface determinants from a wide range of settings/populations that appear less likely to be identified *in vivo*. Indeed, RES was the only method that surfaced an outer setting determinant. Researchers may wish to consider the insights gained by sequencing methods. For example, by leveraging RES first to ground an ethnography in theory and the “bigger picture.”

### Strengths and Limitations

Strengths include direct comparison of emerging methods and the innovative framing of a “head-to-head” comparison of methods. Limitations include reliance on rapid approaches that may trade depth for feasibility, variation in sample size across methods (e.g., number of informants vs. number of articles), and the fact that this study was done in a geographically restricted area, which could limit generalizability.

## Conclusions

Multi-method determination evaluations can provide rich, nuanced insights in relation to single-method approaches. All three determinant identification methods surfaced implementation determinants that were important in understanding the implementation of MBC. While REA, probes, and RES each surfaced overlapping determinants, REA and RES specifically surfaced unique determinants. Future research should continue building on this work to understand how the combination or sequencing of methods influences the development of tailored implementation strategies and implementation success.

## Supplementary Material

Supplementary Files

This is a list of supplementary files associated with this preprint. Click to download.

- AdditionalFile1.SearchCriteriaandSearchStrings.pdf
- AdditionalFile2.DesignProbeActivityCards.pdf

## Figures and Tables

**Table 1:** Determinants by Ecological Level and Identification Method

CFIR Domain	Method			Determinant
	REA	RES	DP	
Individual (patient)	1	1	1	Resistance to or concerns with completing measures
	1	0	1	Lack of clarity on measures/MBC process
	1	1	0	Clinical presentation
	0	1	0	Insurance status
	0	1	0	Attendance
	0	1	0	Turnover
	1	0	0	Youth patients do not want to involve parents in treatment
Individual (clinician/staff)	1	1	1	Attitudes toward measures, MBC
	1	1	1	Knowledge, self-efficacy, and skill
	1	0	1	Avoidance/anxiety/discomfort related to MBC
	1	1	1	Competing demands/lack of time in session
	1	0	1	Clinicians/staff forget to administer or review measures
	1	1	0	Resistance to change
	1	1	0	Stress & burnout
	0	1	0	Clinicians decide who does/does not get MBC
Inner Setting	1	1	1	Organizational norms, culture, and climate
	1	1	1	General administrative burden (competing demands outside of session)
	1	1	1	Lack of built-in time to do administrative MBC (review scores)
	1	0	1	Inconsistent measurement/documentation of measurement
	1	0	1	Clinicians have variable access to measures
	1	1	1	Lack of reminders
	1	1	0	System/Organization lacks clarity on administrative or systemic utility of MBC
	1	1	0	Clinician/staff turnover
	1	0	0	No discussions within or between teams about MBC process
	1	0	1	Intra-agency cross talk re: policies, procedures
	1	1	0	MBC data are challenging to aggregate at the organizational level
	1	1	0	Administrative leadership support
	0	1	0	Clinics lack technological infrastructure to support MBC or MBC program
	0	1	0	Low baseline MBC use
Outer Setting	0	1	0	Organizations do not have guidance on selecting standardized self-report measures
Implementation Process	1	1	1	No initial or continued training on measures, the MBC process, and the MBC system
	1	1	1	Lack of centralized policies, procedures, and communications related to MBC
	1	0	0	Therapists do not receive feedback on MBC (supervision or administrative)
	1	0	1	Logistical challenges for providers who are not onsite or embedded elsewhere (e.g., CMHC clinicians embedded in a school)
	1	1	0	Lack of incentives
Innovation	1	1	1	MBC system or workflow is cumbersome to patients and/or clinicians
	1	1	1	Cultural and linguistic relevance of measures
	1	1	1	Clinicians and/or patients cannot easily access measure scores/item scores/clinical cutoffs
	1	1	1	MBC is a complex process with a steep learning curve
	1	0	1	Measure construction/quality
	1	0	1	Utility of screening tools as outcome questionnaires
	1	0	0	Frequent changes to MBC process/system

*Note*. CFIR = Consolidated Framework for Implementation Research. REA = Rapid Ethnographic Assessment. RES = Rapid Evidence Synthesis. DP = Design Probes.

## Abbreviations

CFIR: Consolidated Framework for Implementation Research
MBC: Measurement-Based Care
NIMBLE: Novel Methods for Implementing Measurement-Based Care in Low-Resource Environments
REA: Rapid Ethnographic Assessment
RES: Rapid Evidence Synthesis
